# Vascular Anatomy in Congenital Lung Lesions—Description and Classification

**DOI:** 10.3389/fped.2022.900538

**Published:** 2022-05-12

**Authors:** Simon Kargl, Florian Schlader, Mario Scala, Julian Kammel

**Affiliations:** ^1^Medical Faculty, Johannes Kepler University Linz, Linz, Austria; ^2^Department of Pediatric Surgery, Kepler University Hospital, Linz, Austria; ^3^Competence Center for Pediatric Radiology, Kepler University Hospital, Linz, Austria

**Keywords:** congenital pulmonary lesion, vascular anatomy, bronchopulmonary sequestration, hybrid lesion, congenital pulmonary airway malformation

## Abstract

**Background:**

Bronchopulmonary sequestration (BPS) and hybrid lesion of congenital pulmonary airway malformation (CPAM) are congenital lung lesions typically presenting with systemic vascular connection. We describe and categorize this atypical systemic vascular anatomy in congenital lung lesions.

**Methods:**

In a medical chart review from 2005 to 2020 patients with systemic vascular connection of congenital lung lesions were identified. Clinical and radiological data were collected and compared. Two experienced pediatric radiologists reviewed postnatal thoracic contrast-enhanced computed tomography scans to describe and categorize atypical vascular anatomy. We completed our findings with a review on vascular anatomy in congenital lung lesions.

**Results:**

A total of 21 patients with congenital lung lesions (nine extralobar BPS, five intralobar BPS, seven hybrid lesions) had systemic arterial supply; with seven of these additionally having systemic venous drainage. Origin of the feeding arteries from the aorta or aortic main branches was described as supra-diaphragmatic (descending thoracic aorta) in nine and infra-diaphragmatic in ten patients (abdominal aorta, celiac trunk). In two patients with hybrid lesions both supra- and infra-diaphragmatic arterial feeders were found. Additional systemic venous connection of supra-diaphragmatic type drains into the azygos-hemiazygos system (4/21) while the infra-diaphragmatic type (3/21) drains into caval vein, portal or splenic vein.

**Conclusion:**

Various variants of systemic arterial and venous connection of congenital lung lesions can be found. Classification of systemic arterial connection as well as venous drainage of congenital lung lesions as supra-diaphragmatic and infra-diaphragmatic types is intuitive, simple and may be important for the surgeon to avoid unanticipated situations and to perform safe resections.

## Introduction

Congenital pulmonary lesions are occasionally detected in prenatal ultrasound examinations. The most common lesions are congenital pulmonary airway malformation (CPAM) and bronchopulmonary sequestration (BPS). Atypical systemic blood supply is a characteristic feature of BPS, but there is also a number of CPAMs presenting with systemic vascular connection referred to as hybrid lesions (1). Prenatal Doppler ultrasound examination may depict systemic feeding vessels and postnatal contrast-enhanced computed tomography scan (CECT) allows precise presentation of vascular anatomy including venous drainage of congenital lung lesions. This knowledge about atypical systemic vascular anatomy is necessary for correct diagnosis and essential to perform save surgical resection of these congenital lung lesions. Although a few reports pay some attention to atypical systemic vessels in congenital lung lesions, it is time for a closer look and a classification of these variants in vascular supply of congenital lung lesions.

## Methods

This is a retrospective chart review performed in a tertiary perinatal center in a large Austrian city. After institutional review board approval, all patients with BPS and CPAM were identified in prenatal, neonatal and surgical database from 2005 to 2020. Only patients with systemic vascular connection (arterial and/or venous) of congenital lung lesion were included. Medical records were reviewed for pre- and postnatal clinical data; complications and outcome of treatment were assessed. Two experienced pediatric radiologists reviewed postnatal CECTs (iodixanol) to precisely describe the atypical anatomy of both arterial and venous systemic connection of congenital pulmonary lesions. We focus on description and categorization of vascular anatomy of these congenital lung lesions. A literature review on vascular characteristics in congenital lung lesions completes our data.

## Results

From 2005 to 2020, 48 patients (22 male/26 female) with either BPS or CPAM were treated at our department of pediatric surgery in a tertiary university hospital. In 39 cases these congenital lung lesions were identified in prenatal ultrasound examination. Postnatal CECT scan did not depict systemic vascular supply in 25 of 48 patients with congenital lung lesions—these were “classical” CPAM. In two patients, prenatally diagnosed congenital lung lesion was not confirmed postnatally: one child had a normal CECT scan and the other showed small vascular connections between the thoracic aorta and the pulmonary artery in the absence of a lung lesion.

In 21 cases postnatal CECT depicted systemic vascular connection of congenital lung lesions: nine extralobar bronchopulmonary sequestrations (EBPS), five intralobar bronchopulmonary sequestrations (IBPS) and seven hybrid lesions. CECT scans were performed with general anaesthesia at a median age of 95 days (range 1–218 days) in all patients. In seven of these 21 patients, systemic vascular supply had been identified prenatally (two hybrid lesions, two IBPS, three EBPS).

**FIGURE 1:**
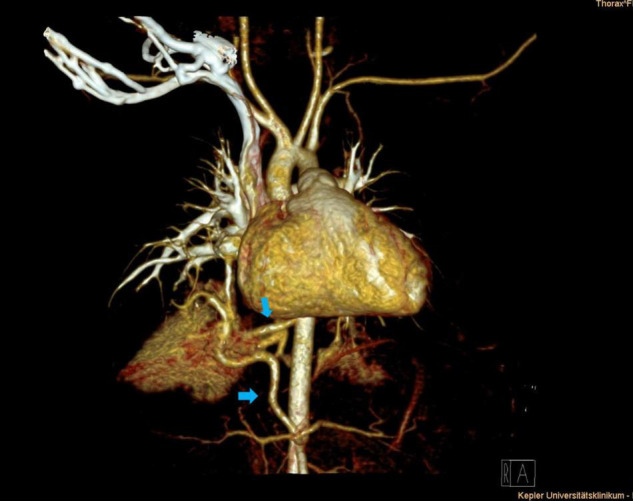
Thoracic contrast-enhanced computed tomography scan (CECT) depicting right lower lobe hybrid lesion. Supra- and infra-diaphragmatic arteries originate from the descending aorta and the celiac trunk (blue arrows).

Arterial blood supply of the congenital lung lesion was categorized as supra-diaphragmatic and infra-diaphragmatic, depending on the origin of the arterial branch (Table 1). Supra-diaphragmatic arterial systemic supply was found in nine patients with the arterial feeder originating from the descending thoracic aorta. Infra-diaphragmatic arterial supply derived from the celiac trunk (*n* = 4) and the abdominal aorta (*n* = 6). Further possible supra- and infra-diaphragmatic types of arterial feeders in congenital lung lesions are presented in Table 2. In five patients congenital lung lesions had at least two arterial feeders: two of them had both supra- and infra-diaphragmatic arterial branches (thoracic descending aorta + celiac trunk) (Figure 1), while the others were supra-diaphragmatic (*n* = 2) or infra-diaphragmatic types (*n* = 1). Maximum diameter of the arterial systemic branches ranged from 1.0 to 5.0 mm (median = 2.3 mm).

**TABLE 1:** Systemic arterial supply of congenital lung lesion.

Origin of arterial feeder	Number of patients (*n* = 21)
Supra-diaphragmatic arterial type	9
• Thoracic descending aorta	9 (4 hybrid lesions, 5 iBPS)
Infra-diaphragmatic arterial type	10
• Abdominal aorta	6 (eBPS)
• Celiac trunk	4 (1 hybrid lesion, 3 eBPS)
Supra- + infra-diaphragmatic type	2
• Thoracic descending aorta + celiac trunk	2 hybrid lesions

**TABLE 2:** Systemic venous drainage of congenital lung lesion.

Anatomy of venous drainage	Number of patients (*n* = 7)
Supra-diaphragmatic venous type	4
• Azygos vein	2 hybrid lesions, 1 eBPS
• Hemiazygos vein	1 eBPS
Infra-diaphragmatic venous type	3
• Inferior vena cava	1 eBPS
• Splenic vein	1 eBPS
• Portal vein	1 eBPS

**FIGURE 2:**
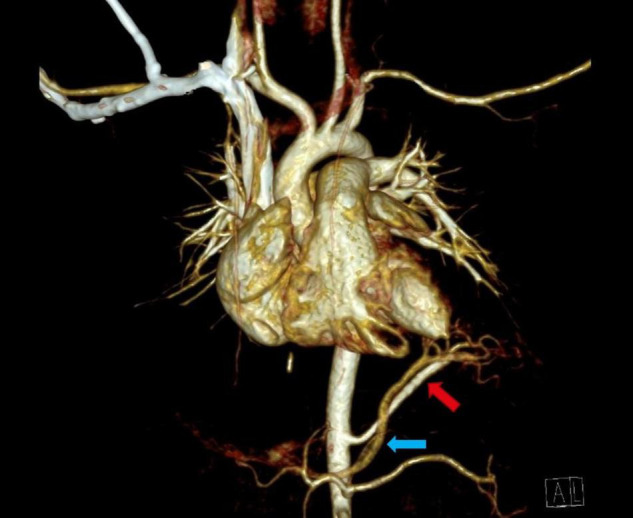
Thoracic contrast-enhanced computed tomography scan (CECT) showing infra-diaphragmatic arterial and venous systemic connection of left inferior extralobar bronchopulmonary sequestration: arterial supply originates from the abdominal aorta (red arrow), venous drainage via portal vein (blue arrow).

Seven of twenty-one cases of congenital lung lesions with systemic arterial supply additionally had atypical systemic venous drainage (Table 3). Supra-diaphragmatic types of venous drainage were found in four and infra-diaphragmatic types in three patients. Supra-diaphragmatic venous types of congenital lung lesions drain via azygos-hemiazygos system while infra-diaphragmatic types use caval vein or other visceral veins for drainage (Figure 2). In all three patients with infra-diaphragmatic venous drainage, systemic arterial connection was also infra-diaphragmatic type. Two patients with supra-diaphragmatic venous type congenital lung lesion had both supra- and infra-diaphragmatic arterial feeders. In one patient congenital lung lesion had two large venous vessels: supra-diaphragmatic venous drainage via azygos vein and an intrapulmonary drainage via pulmonary vein. All other 14 lesions with systemic arterial supply had exclusive venous drainage via the pulmonary veins, including all the five cases of intralobar BPS. Maximum diameter of the venous systemic branches ranged from 1.2 to 6.0 mm (median = 2.7 mm).

**TABLE 3:** Supra-diaphragmatic and infra-diaphragmatic origin of arterial feeders in congenital lung lesions (2–7).

Supra-diaphragmatic type	Infra-diaphragmatic type
• Thoracic aorta	• Abdominal aorta
• Subclavian artery	• Celiac trunk
• Intercostal arteries	• Superior mesenteric artery
• Thoracic internal artery	• Left gastric artery
• Pericardiophrenic artery	• Splenic artery
	• Suprarenal artery
	• Renal artery

Open surgical resection of BPS and hybrid lesion was performed in all 21 patients with congenital lung lesion and systemic vascular supply. Postoperative complications were found in three patients (3/21). In two patients pneumothorax urged chest tube insertion in the postoperative phase. One patient had postoperative hemothorax due to blood oozing from injured intercostal vessels necessitating revision surgery within 8 h. No bleeding occurred from the ligated atypical systemic arteries or veins. At follow up 6 months after surgery all patients presented without any complaints.

## Discussion

In congenital lung lesions, systemic vascular connection of the lesion is a frequent and characteristic finding. Atypical arterial and venous vessels play an important role in diagnosis and classification of these lesions. BPS may be diagnosed prenatally by depiction of a lung mass with systemic arterial connection. It is well-known that intralobar and extralobar bronchopulmonary sequestration (IBPS, EBPS) may be distinguished by venous return of the lesion: IBPS drains via pulmonary veins while EBPS drains systemically. An overlap between BPS and CPAM has been found and this congenital lung lesion has been described as hybrid lesion—a CPAM with systemic vascular connection (1). It is noteworthy that, atypical systemic arterial supply to parts of the lung may also occur in the absence of congenital lung lesions. In these rare cases, atypical systemic arteries supply normal basal segments of the right or left lung (2, 3).

Systemic arterial supply in congenital lung lesions seems to frequently originate from the thoracic or abdominal aorta (4). Nevertheless, arterial supply may come from various supra- and infra-diaphragmatic aortic branches. In congenital lung lesions in the upper thoracic region the surgeon must be aware of possible arterial connection to the subclavian artery (4, 5). As represented in our series, infra-diaphragmatic arterial blood supply regularly comes from the celiac artery, but origin from other visceral arteries has also been described: superior mesenteric artery, left gastric, splenic artery and even suprarenal or renal artery (4, 7–9). Hou et al. recently published a series of 23 adult patients with BPS and anomalous systemic arteries originating from the descending thoracic aorta in all cases without any infra-diaphragmatic arterial supply (10). Nevertheless, infra-diaphragmatic systemic blood supply of extralobar and even intralobar BPS can be found (4, 7). Long et al. described in their retrospective CT angiography study the interesting finding that all feeding arteries in left sided basal pulmonary sequestration came from the thoracic aorta; our series did not corroborate this finding.

In our series five patients had more than one systemic artery. As described in the sparse literature on this subject, this may not be a rare finding (11) and even three separate feeding arteries have been reported in congenital lung lesions (12).

The most distal systemic arterial feeder in our series originated from the celiac trunk; but even arterial supply coming from a renal artery has been described (7). In this context, an autopsy study from Stocker et al. is notable: In children without congenital lung lesions or vascular disease, they demonstrated systemic arteries in the pulmonary ligament in 10 of 11 cases arising from the thoracic aorta (13).

Atypical systemic venous drainage occurs less frequently than atypical arterial supply in congenital lung lesions, as the majority of intralobar BPS show normal venous drainage via pulmonary veins. Nevertheless, systemic venous drainage may also be found in IBPS (6). Supra-diaphragmatic systemic venous drainage in congenital lung lesions seems to be frequently connected to the azygos and less commonly to the hemiazygos system (4, 7–9). Supra-diaphragmatic systemic venous return into the subclavian vein (6) and intercostal vein (9) has been described. Infra-diaphragmatic systemic venous drainage, as in one of our patients with EBPS, has been found via portal vein (9, 14–17), inferior caval vein, splenic and suprarenal vein. Congenital lung lesions with both infra-diaphragmatic visceral inflow and infra-diaphragmatic outflow have been described (14). Interestingly, we found that all patients with infra-diaphragmatic type venous drainage also had infra-diaphragmatic type systemic arterial connection. In contrast to systemic arterial supply, a congenital lung lesion with both supra-diaphragmatic and infra-diaphragmatic type venous drainage has not been described. It is noteworthy, that isolated systemic venous connection of a congenital lung lesion in the absence of systemic arterial supply has neither been found in our series nor did we find any cases in medical literature.

In congenital lung lesions, atypical systemic vascular connection is a major cause of morbidity: high output cardiac failure, congestive heart failure or hemoptysis may occur (18). Although rare, unanticipated injury to arterial feeding vessels in surgery for congenital lung lesions may cause massive hemorrhage (19). Knowledge of vascular anomalies in congenital lung lesions is therefore essential for pediatric surgeons caring about these patients.

## Conclusion

The high degree of variability of systemic vascular anatomy necessitates clear and intuitive classification of this systemic arterial and venous connection in congenital lung lesions. Based on the origin of systemic arteries and veins we differentiate supra-diaphragmatic and infra-diaphragmatic vascular types of congenital lung lesions. This differentiation has practical-surgical relevance: as the diaphragm separates abdominal and thoracic cavity, vascular injury during surgery in congenital lung lesion with infra-diaphragmatic arterial supply may cause fatal intraabdominal bleeding. Therefore, our classification may contribute to correct preoperative evaluation to avoid unanticipated vascular complications.

## Data Availability Statement

The original contributions presented in the study are included in the article/supplementary material, further inquiries can be directed to the corresponding author.

## Author Contributions

SK: study conception and design. FS, MS, and JK: data collection. SK, FS, MS, and JK: analysis and interpretation of results and draft manuscript preparation. All authors reviewed the results and approved the final version of the manuscript.

## Conflict of Interest

The authors declare that the research was conducted in the absence of any commercial or financial relationships that could be construed as a potential conflict of interest.

## Publisher’s Note

All claims expressed in this article are solely those of the authors and do not necessarily represent those of their affiliated organizations, or those of the publisher, the editors and the reviewers. Any product that may be evaluated in this article, or claim that may be made by its manufacturer, is not guaranteed or endorsed by the publisher.
